# Post-conventional moral reasoning is associated with increased ventral striatal activity at rest and during task

**DOI:** 10.1038/s41598-017-07115-w

**Published:** 2017-08-02

**Authors:** Zhuo Fang, Wi Hoon Jung, Marc Korczykowski, Lijuan Luo, Kristin Prehn, Sihua Xu, John A. Detre, Joseph W. Kable, Diana C. Robertson, Hengyi Rao

**Affiliations:** 1 Laboratory of Applied Brain and Cognitive Sciences, Shanghai International Studies University, Shanghai, China; 2 Center for Functional Neuroimaging, Department of Neurology, University of Pennsylvania, Philadelphia, United States of America; 3 Department of Psychology, University of Pennsylvania, Philadelphia, United States of America; 4 Department of Legal Studies & Business Ethics, The Wharton School, University of Pennsylvania, Philadelphia, United States of America; 5 Department of Neurology & NeuroCure Clinical Research Center, Charité Universitätsmedizin Berlin, Berlin, Germany

## Abstract

People vary considerably in moral reasoning. According to Kohlberg’s theory, individuals who reach the highest level of post-conventional moral reasoning judge moral issues based on deeper principles and shared ideals rather than self-interest or adherence to laws and rules. Recent research has suggested the involvement of the brain’s frontostriatal reward system in moral judgments and prosocial behaviors. However, it remains unknown whether moral reasoning level is associated with differences in reward system function. Here, we combined arterial spin labeling perfusion and blood oxygen level-dependent functional magnetic resonance imaging and measured frontostriatal reward system activity both at rest and during a sequential risky decision making task in a sample of 64 participants at different levels of moral reasoning. Compared to individuals at the pre-conventional and conventional level of moral reasoning, post-conventional individuals showed increased resting cerebral blood flow in the ventral striatum and ventromedial prefrontal cortex. Cerebral blood flow in these brain regions correlated with the degree of post-conventional thinking across groups. Post-conventional individuals also showed greater task-induced activation in the ventral striatum during risky decision making. These findings suggest that high-level post-conventional moral reasoning is associated with increased activity in the brain’s frontostriatal system, regardless of task-dependent or task-independent states.

## Introduction

Most people have strong moral beliefs about what is right and wrong. Although the existence of moral values is universal across different cultures and ethnic groups, people vary in their moral development (also called moral reasoning), that is, the ability to analyze complex moral issues^1^. Kohlberg’s seminal theory^1^ is the most influential and widely researched theory of moral reasoning and moral development. Even today more than twenty-five years after Kohlberg’s death, there is considerable research conducted using his theory. Much of this influence can be attributed to this theory’s central and much-tested assumption that an individual’s moral reasoning will predict moral behavior. To capture such individual differences in moral development, Kohlberg’s theory^1^ classified moral development into three levels: pre-conventional level (motivated by self-interest); conventional level (motivated by maintaining social-order, rules and laws); and post-conventional level (motivated by social contract and universal ethical principles). These three levels of moral reasoning can be objectively assessed by the Defining Issues Test (DIT-2) developed by Rest and colleagues^2^ in terms of preferences for specific cognitive schemas (see Material and Methods for a detailed explanation). Based on this theory, the DIT is the most widely used instrument in moral development research^3^ and the best documented in terms of reliability and validity^4^. A revised version of the DIT, known as the DIT-2^2^, makes several improvements, including updating dilemmas and items, constructing a new index (called N2 score which reflects the extent to which a person prefers post-conventional moral thinking), and detecting unreliable participants. In addition, the DIT-2 is highly correlated with the DIT (r = 0.79)^2^ and has been used to investigate links with brain structure and function^5, 6^. However, the underlying reasons why people reach different moral development levels are still unclear. In our current study, we aim to explore the possible neural basis of individual differences in moral development.

At the psychological level, researchers have explored links between moral judgment and decision making^7–10^. Moral judgment and decision making share several common components, such as value representations and reinforcement learning. Specifically, Cushman (2013) has argued that action-based and outcome-based values are two important representations in a dual-system framework used to understand diverse moral judgments^10^. According to this framework, the motivations for moral judgment can be valuing the intrinsic status of actions (e.g., I don’t steal bread because theft is wrong) or valuing the expected consequences of actions (e.g., “If I steal bread, it will cause the owner harm”). Previous studies^11, 12^ have demonstrated that normal controls are able to pair the representations of the transgressive action with the distress cues of the victim (i.e., make reference to another’s pain) through classical conditioning. However, psychopaths fail to establish this conditioning to make reference to another’s pain, suggesting the important role of value-based reinforcement learning in moral reasoning development in situations such as avoiding outcomes that might hurt others.

Reward motivation is another possible explanation of individual differences in moral development. According to conventional economic theories of decision making, people aim to maximize subjective expected values, but differ with regard to preferences for given options (e.g., taking or avoiding risks)^13^. Accumulating evidence also suggests a relationship between moral reasoning and prosocial behaviors oriented towards the common good^4^, showing that individuals with high-level moral reasoning are more likely to engage in moral and prosocial behaviors^14^, albeit not always^15^. Although the mechanisms underlying prosocial behaviors remain controversial, two competing theories exist. One theory suggests that prosocial behaviors, regardless of the presence/absence of social pressure, can be guided by the objective of maximizing the intrinsic value (moral value) placed on social principles (e.g., fairness and cooperation). An alternative theory proposes that any actions of prosociality under social pressure are selfishly motivated to protect one’s reputation or avoid others’ resentment. According to Kohlberg’s moral development theory, individuals who reach the post-conventional level live by their own ethical principle, including basic human rights as life, liberty, and justice. They tend to pursue “the greatest good for the greatest number of people”^1^.Thus, it raises the possibility that differences in reward motivation for prosocial behaviors between individuals are associated with different moral development level. It is conceivable that higher level moralists perform prosocial behavioral based on their own ethical principles instead of protecting their reputations or avoiding others’ resentment.

At the neural level, both value-based reinforcement learning and prosocial behaviors engage overlapping neural systems with moral judgment. Decades of neuroimaging research have identified multiple brain regions associated with moral judgment, including the dorsolateral prefrontal cortex (DLPFC), ventromedial prefrontal cortex (vmPFC), posterior cingulate cortex, superior temporal cortex, temporal poles, temporoparietal junction, striatum, amygdala, and insula^16–18^. Furthermore, recent studies have provided evidence that subjective value, in both moral and non-moral contexts, is represented in a domain-general manner, in ventral striatum (VS) and vmPFC^19–22^. For instance, Shenhav and Greene^22^ showed that activations in the VS and vmPFC were correlated with the expected moral value of actions (i.e., the expected number of lives lost/saved) during hypothetical life-and-death moral dilemmas. Most of these regions are also utilized in value-based decision making. Specifically, a “reward-network” composed of the striatum and vmPFC subserves affective/incentive processing of rewards and punishments during decision making^8, 23–25^. Additionally, prosocial behaviors are also mediated by these value-based brain regions. For example, increased activation in the VS and vmPFC has been observed when individuals make charitable donations and altruistic decisions in a similar manner to that observed when individuals receive monetary rewards^19, 26, 27^. Taken together, these findings suggest the involvement of the mesolimbic frontostriatal value-based processing system in moral reasoning. Remarkably, we recently found an association between high-level post-conventional moral reasoning and increased gray matter volume (GMV) in vmPFC/subgenual ACC ﻿(sgACC)^5^. This work established a link between moral reasoning and brain structure in the frontostriatal system, suggesting that the frontostriatal network may be one of the key neural bases of individual differences in moral development. Here, we further investigate whether this link extends to the function, in addition to the structure, of the brain’s frontostriatal reward system.

To directly test this association, in the present study we examined a sample of 64 Wharton School Master of Business Administration (MBA) students using functional MRI (fMRI) during a resting state without task requirements, as well as during the performance of a monetary risky decision making task. For the resting state, we used arterial spin labeling (ASL) perfusion fMRI to quantify resting neural activity (i.e., cerebral blood flow, CBF) in the absence of task requirements. We hypothesize that individuals with different levels of moral development will show different resting CBF in the frontostriatal system.

For the task state, we used blood oxygen level-dependent (BOLD) fMRI to examine the task-induced brain response during the Balloon Analogue Risk Task (BART)^28^. We chose the BART task for several reasons. First, previous studies have consistently shown that the BART task can induce reliable and robust activation in the frontostriatal reward processing system^28–31^, which is involved in both moral judgment and value-based decision making. Thus, we use the BART task with fMRI to examine whether task-induced brain activation in the frontostriatal reward processing system differs according to moral development levels. Second, the relationship between moral development and risk taking behaviors remains controversial^32^. Some studies argue that individuals tend to view risky behaviors (i.e., substance use, suicide, sexual involvement) as a matter of personal choice instead of a moral issue^33, 34^, and moral reasoning does not consistently predict risky behaviors, such as substance involvement^35–37^. However, other studies have observed the association between lower moral reasoning and higher drug usage, especially in adolescents^38, 39^. The BART is able to provide an ecologically valid model for the assessment of risk taking propensity, and performance on the BART has been shown to correlate with real-world risk-taking and impulsive behaviors^40–43^. Thus we use the BART performance to examine the relationship between moral development and risk taking behaviors. We aim to explore whether moral development will modulate both risk taking behavior and frontostriatal activation during the BART.

## Results

### Demographic information and personality traits

The participants included 38 students who had reached the level of post-conventional moral reasoning (post-con group) and 26 students who were at the pre-conventional or conventional moral reasoning level (pre-con + con group), as defined by the DIT-2. These two groups did not differ in age or gender (p > 0.9; Table 1). With regard to the five personality dimensions measured by the NEO-PI-R, the post-con group, compared to the pre-con + con group, had higher scores in Openness to Experience (63.84 vs. 54.62, p = 0.001) and lower scores in Neuroticism (48.58 vs. 54.15, p = 0.02).

**Table 1:** Demographic information, personality scores, and behavioral performances.

Variables	Post-con group (n = 38)	Pre-con + con group (n = 26)	Statistics
	Mean (SD)	Mean (SD)	p-value
Gender (N, male/female)	21/17	14/12	0.91
Age (years)	27.11 (1.67)	27.12 (1.63)	0.98
DIT-N2 score	51.16 (10.56)	30.70 (9.89)	p < 0.001
Personality traits^1^			
Openness	63.84 (11.42)	54.62 (9.01)	0.001
Conscientiousness	52.45(9.07)	53.04(11.99)	0.82
Extraversion	53.84(9.54)	56.65(14.30)	0.35
Agreeableness	48.89(11.05)	44.65(12.16)	0.15
Neuroticism	48.58(9.58)	54.15(9.57)	0.02
BART Behavioral data			
Total trials (number of balloons)	26.03 (3.72)	26.04 (4.42)	0.49
Win trials	16.79 (4.77)	16.82 (5.54)	0.20
Loss trials	9.24 (2.95)	9.21 (3.57)	0.19
Pumps for each balloon	7.01 (1.17)	6.95 (1.01)	0.18

Post-con group, post-conventional group; Pre-con + con group, pre-conventional and conventional group; DIT, Defining Issues Test; BART, Balloon Analogue Risk Task.

^1^Personality scores were assessed by NEO-PI-R. Two pre-con + con subjects’ data were not available due to technical reasons.

### Resting neural activity

**Figure 1:**
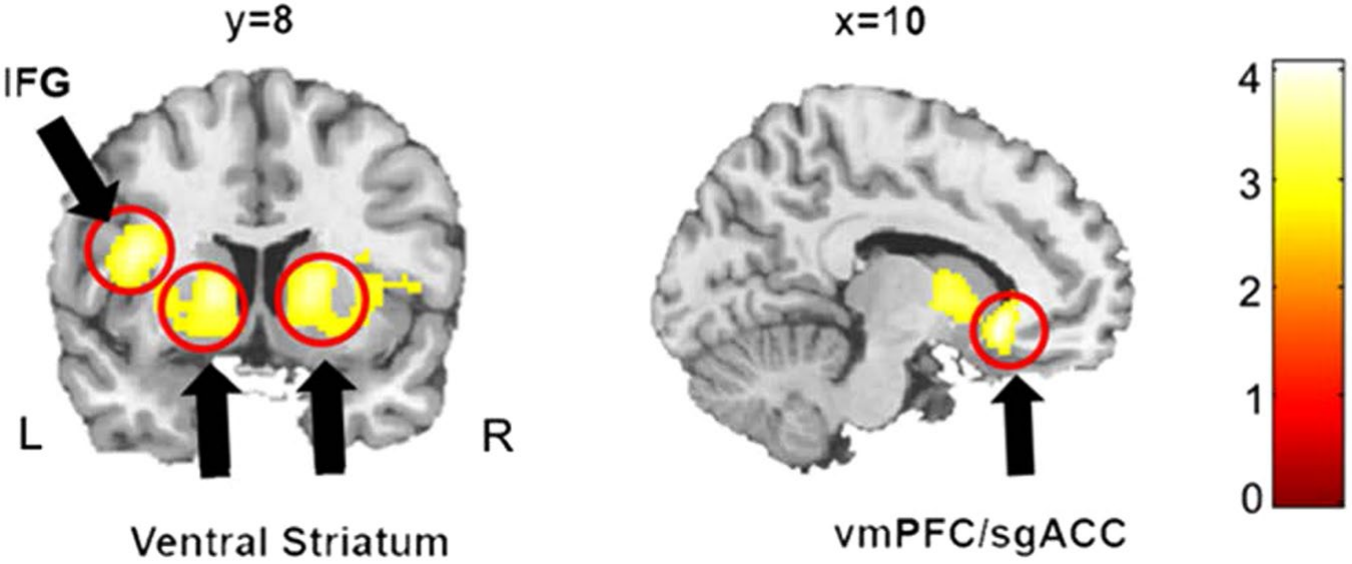
Differences in resting CBF between the post-con group and the pre-con+con group at whole brain level. The post-con group showed higher CBF values in the bilateral ventral striatum (VS), the ventromedial prefrontal cortex/subgenual anterior cingulate cortex (vmPFC/sgACC), and the inferior frontal gyrus (IFG/AIS) compared to the pre-con+con group. All regions survived whole brain cluster-level FWEcorrection at p < 0.05.

Controlling for variables showing significant group differences (i.e., Openness and Neuroticism), the post-con group showed increased CBF in bilateral VS, mainly located in the nucleus accumbens extending to putamen and caudate (peak x, y, z coordinates = [−16, 8, 8]; [16, 6, 8]), vmPFC extending to subgenual ACC (vmPFC/sgACC; peak x, y, z coordinates = [10, 24, −8]), and inferior frontal gyrus adjacent to anterior insula (IFG/AIS; peak x, y, z coordinates = [−40, 10, 20]) compared to the pre-con + con group. All these clusters survived at whole brain cluster-level family-wise error (FWE) correction at *p* < 0.01. These results are illustrated in Fig. 1 and Table 2.

**Table 2:** Brain regions showing increased CBF in the post-con compared to the pre-con + con group.

Regions	MNI Coordinate			Peak z scores	Cluster-level FWE corrected	Cluster size
	X	Y	Z		p-value	
L. Ventral striatum	−16	8	8	3.54	0.001	2,755
R. Ventral striatum	16	6	8	3.49	0.001	
R. vmPFC/sgACC	10	24	−8	3.77	0.001	
L. Inferior frontal gyrus/anterior insula	−40	10	20	3.59	0.009	1,781

L, left; R, right; vmPFC/sgACC, ventromedial prefrontal cortex/subgenual anterior cingulate cortex.

### Association between resting neural activity and the extent of moral development

**Figure 2:**
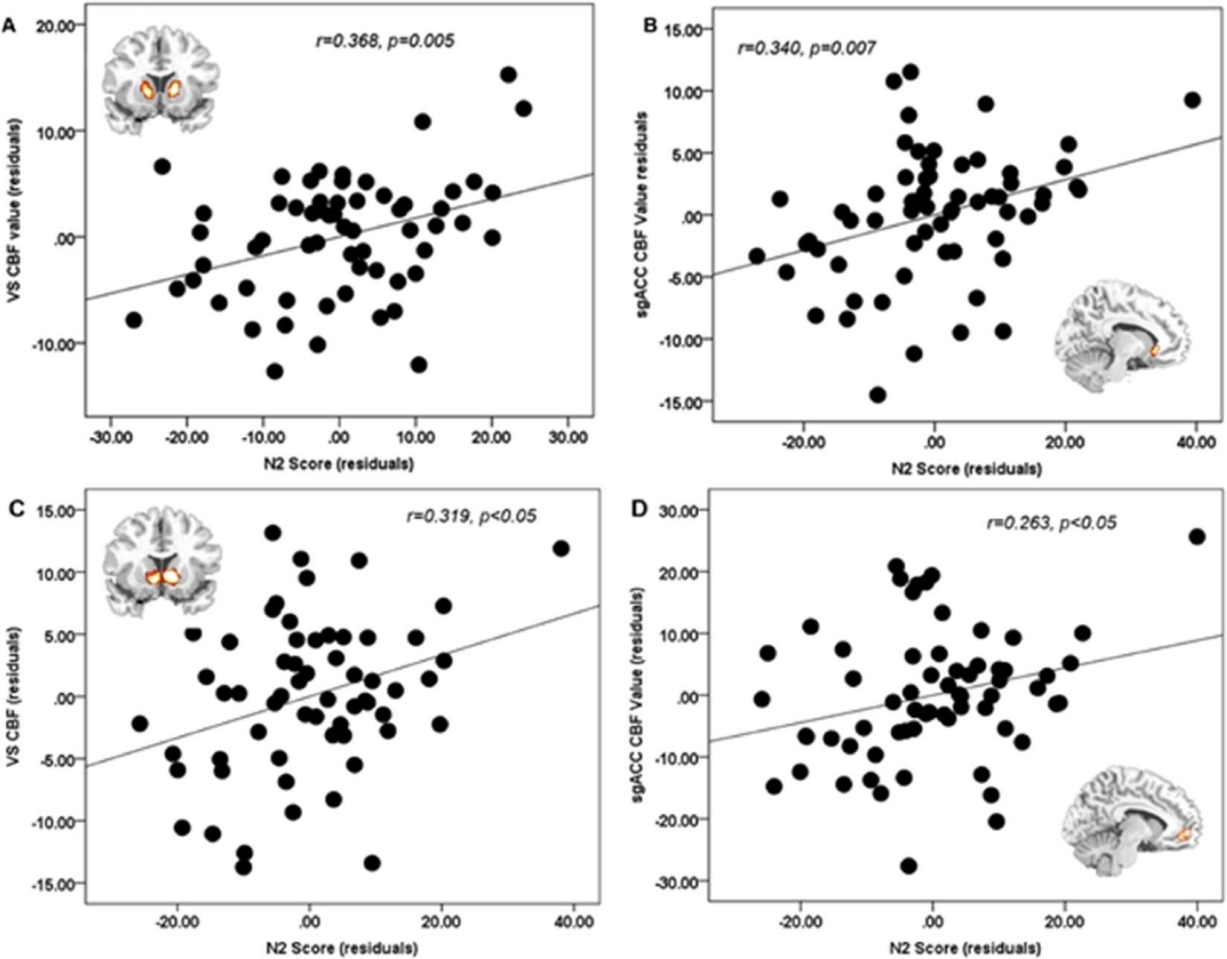
Association between resting CBF values and DIT-N2 Scores. Resting CBF values in (**A**) ventral striatum and (**B**) ventromedial prefrontal region were positively correlated with DIT-2 N2 scores. The functional ventral striatum (VS) ROI and the ventromedial prefrontal ROI were defined based on the CBF results. Independent ROI analyses revealed similar results that (**C**) VS and (**D**) ventromedial prefrontal regions were positively correlated with DIT-2 N2 scores. The independent VS ROI and ventromedial prefrontal ROI were defined based on previous literature^24^. Openness and Neuroticism were controlled in the partial correlation analyses.

Partial correlation analyses (controlling for Openness and Neuroticism) were conducted to illustrate the association between resting CBF in the VS, vmPFC/sgACC, and IFG/AIS regions and the DIT-2 N2 score, an index describing the extent to which a person exhibits post-conventional moral thinking. For this analysis, functional regions-of-interest (ROIs) for VS, vmPFC/sgACC, and IFG/AIS were derived from the resting CBF analysis described above. DIT-2 N2 scores were positively correlated with resting CBF values in VS (r = 0.386, *p* = 0.005) and vmPFC/sgACC (r = 0.340, *p* = 0.007; Fig. 2), but not with those of IFG/AIS (*p* > 0.1). No correlations were observed between resting CBF and the personality variables (Openness and Neuroticism, both *p > *0.2). To validate the association between DIT-2 N2 scores and resting CBF (i.e., VS and vmPFC/sgACC) in independently defined ROIs, and confirm that these two regions defined by our CBF analysis are consistent with the regions sensitive to reward valuation and subjective value across prior studies, we conducted additional analyses with ROIs defined from a previous meta-analysis study by Bartra *et al*.^24^. The additional partial correlation analysis revealed similar results, i.e., that DIT-2 N2 scores were positively correlated with resting CBF values in the VS (r = 0.319, *p < *0.05, Fig. 2C) and vmPFC/sgACC (r = 0.263, *p* < 0.05, Fig. 2D).

### Relationship between the vmPFC/sgACC gray matter volume and resting CBF

In our previous study from the same cohort^5^, the high-level post-conventional group showed increased gray matter volume (GMV) in vmPFC/sgACC compared to the low-level pre-conventional + conventional group. Given the functional connectivity between the vmPFC and ventral striatum^44, 45^, we examined if the GMV difference in vmPFC/sgACC influenced brain activation differences between the two DIT groups. Controlling for personality traits and vmPFC/sgACC GMV, DIT group still had a significant main effect on resting CBF in the VS (*t* (59) = 3.33, *p* = 0.001) and the vmPFC/sgACC (*t* (59) = 3.08, *p* = 0.003). No significant correlation was found between vmPFC/sgACC GMV and resting CBF (all ps > 0.05). These results indicate that the gray matter volume difference between two DIT groups did not influence the resting CBF differences between the two groups.

### Behavioral data during the BART

The post-con and pre-con + con groups did not differ in BART performance (Table 1, all *ps* > 0.1). The post-con group and pre-con + con group completed 26.03 ± 3.72 and 26.04 ± 4.42 balloons, respectively. For each balloon, the post-con group inflated 7.01 ± 1.17 times and the pre-con + con group inflated 6.95 ± 1.01 times. Participants showed more win trials than loss trials across both groups: the post-con group won 16.79 ± 4.77 trials and lost 9.24 ± 2.95 trials (*p* < 0.001); the pre-con + con group won 16.82 ± 5.54 trials and lost 9.21 ± 3.57 trials (*p* < 0.001). No interactions were observed between DIT groups and outcomes (*p* > 0.8).

### Brain activations associated with risk level during the BART

**Figure 3:**
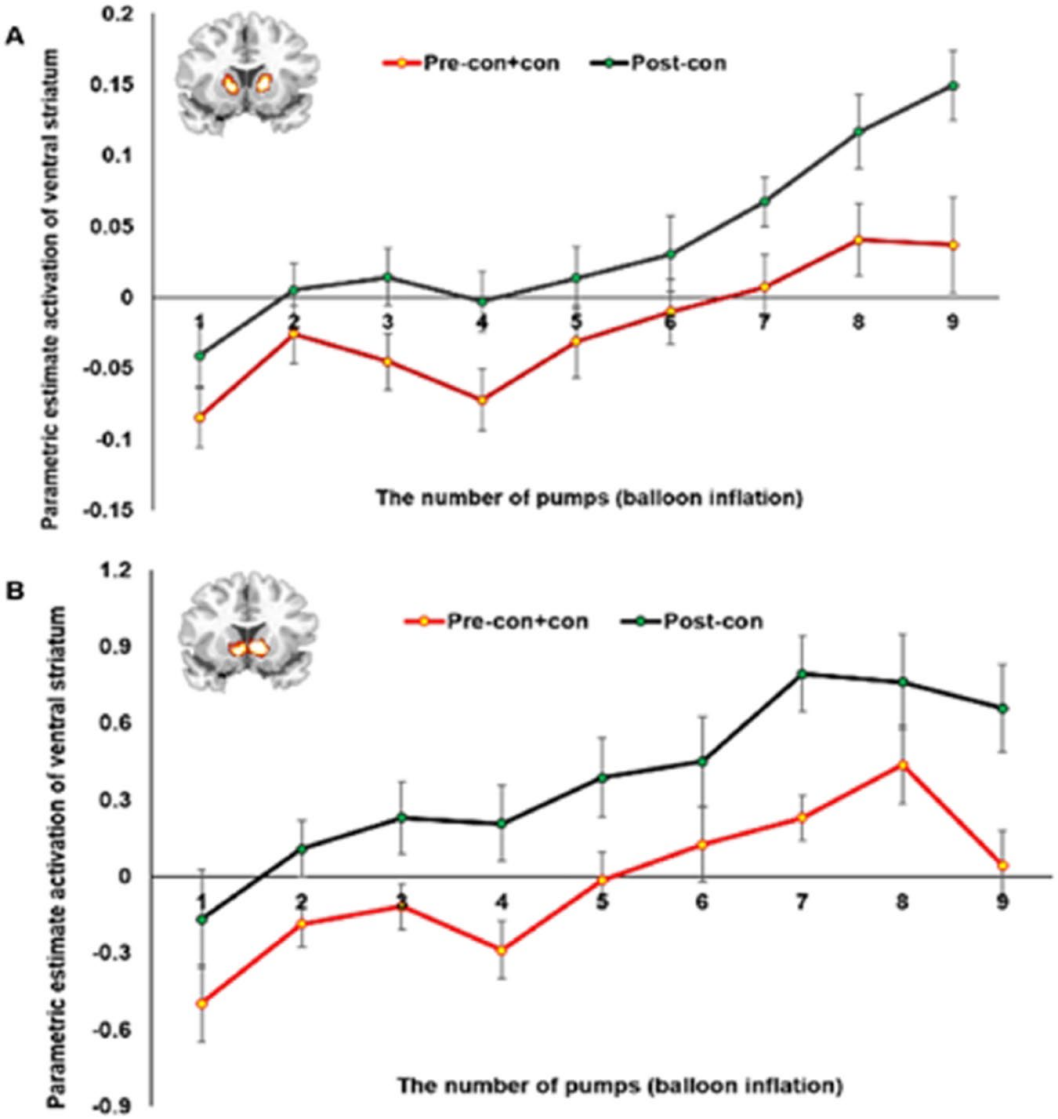
Ventral striatal activation associated with gradually increased risk level in both groups during the BART. (**A**) The functional ventral striatum (VS) ROI was defined based on the CBF results and used to extract VS activation associated with the 9 risk levels. Both groups showed increased brain activation in VS associated with higher risk levels. The post-con group consistently had greater activation across all risk levels (range 1 to 9) compared to the pre-con+con group, and a repeated measures ANOVA revealed a significant main effect between the two groups (F = 5.492, p = 0.018). (**B**) The independent VS ROI was defined based on previous literature^24^ and used for an additional ROI analysis. This additional analysis revealed similar results. A repeated measures ANOVA revealed a significant main effect between the two groups (F = 5.371, p = 0.024).

The level of risk during the BART increases with the number of balloon inflations. That is, while the first inflation of a balloon has the smallest risk of explosion, each additional balloon inflation increases the probability of the balloon popping. To explore the neural correlates associated with risk perception, we analyzed brain activation that co-varied with the gradually increasing level of risk during the BART (i.e., each balloon pump). As mentioned before, we were primarily interested in the relationship between VS and vmPFC function and moral reasoning and thus we focused on activations in these two regions. Using the MarsBaR toolbox (http://marsbar.sourceforge.net/), we calculated parametric estimate values in response to each risk level in the VS and vmPFC/sgACC ROIs defined from the above CBF comparisons. As illustrated in Fig. 3A, compared to the pre-con + con group, the post-con group showed higher activation over all risk levels (range 1 to 9). A repeated measures ANOVA (2 DIT groups and 9 risk levels) revealed a significant main effect of DIT group, indicating increased activity in VS (*F* = 5.492, *p* = 0.018) in the post-con group, and a significant main effect of risk level (*F* = 15.818, *p* < 0.001), indicating greater VS activity associated with the higher risk level. No interaction effect was found between DIT group and risk level (*p* > 0.3). In contrast to the VS, the vmPFC/sgACC did not show any activation differences between the two groups (*p > *0.05).

To confirm our findings, we performed an additional analysis using the VS ROI previously identified as sensitive to reward valuation and subjective value in a meta-analysis study by Bartra *et al*.^24^. This additional analysis revealed similar results. As illustrated in Fig. 3B, both DIT groups showed activation in the independent VS ROI associated with higher risk levels. Compared to the pre-con + con group, the post-con group showed higher activation across all risk levels (range 1 to 9). Consistent with this, a repeated measures ANOVA (2 DIT groups and 9 risk levels) revealed a significant main effect of the DIT group (*F* = 5.371, *p* = 0.024), and a significant main effect of the risk level (*F* = 19.575, *p* < 0.001). No interaction effect was found between the DIT group and the risk level (*p* > 0.5).

As seen in Fig. 3, although the post-con group showed greater BART VS activation relative to the pre-con + con group, the patterns of activation as a function of risk level (range 1 to 9) are very similar across the two groups. That is, if the degree of differences in BOLD responses amplitudes (beta weights of GLM fit) between the two groups is parallel across the entire risk level, BOLD responses between the two groups would be expected to be highly correlated. Consistent with this expectation, an additional correlation test revealed that the VS responses in the post-con group were strongly and linearly correlated with those of the pre-con + con group (Figure S1). In other words, although BOLD response level in the VS differed between the two groups, changes of activation in response to the increasing level of risk were similar. These findings suggest that the increased VS activation in the post-con group during the BART is likely to be intrinsic rather than specific to a certain risk level.

### Relationship between the vmPFC/sgACC gray matter volume and BART activation

Similar to the CBF analysis, we also examined if the GMV difference in vmPFC/sgACC influenced risk level-associated brain activation differences between the two DIT groups. Controlling for personality traits and vmPFC/sgACC GMV, DIT group still had a significant main effect on BOLD activation (average of brain activations associated to all 9 risk levels) in the VS (*t* (59) = 2.71, *p* = 0.009), while we found no such significant main effect of DIT group for the BOLD activation in the vmPFC/sgACC (*t* (59) = 0.91, *p* > 0.05). No significant correlations were found between vmPFC/sgACC GMV and BOLD activation levels during the BART (all ps > 0.05). These results indicate that the gray matter volume difference between two DIT groups did not influence the BART activation differences between the two DIT groups.

### Whole brain activations during the BART and overlaps with the CBF findings

For exploratory purposes to confirm that the BART induced reliable activations in the mesolimbic reward system, we additionally examined whole brain activation patterns in response to the overall risk level for both groups. We observed very similar brain activation patterns across groups (Figure S2). Consistent with previous studies^28, 29^, in both groups increasing risk level was associated with an increase in the activation of mesolimbic frontostriatal regions, including the striatum, vmPFC/sgACC, and midbrain, as well as increased activation in anterior insula, DLPFC, and visual areas. However, direct comparisons between the two DIT groups revealed no activation differences. Additionally, we examined the overlap of VS activation across resting CBF and BOLD activation in the BART. Results showed that the VS region showing CBF differences (Post-con vs. Pre-con + con) overlapped substantially with the VS region showing activation in the post-conventional group and pre-conventional + conventional group during the BART (Figure S3).

## Discussion

The present study characterized differences in resting and task-evoked brain activity associated with different levels of moral reasoning. Significant brain activity differences were observed between individuals at high-level post-conventional moral reasoning and individuals at the pre-conventional and conventional level of moral reasoning. Specifically, using ASL fMRI to quantify regional brain function at rest, we found greater activity in the VS and vmPFC/sgACC in the post-con group compared to the pre-con + con group, and a positive correlation between the activity in these two regions and the degree of post-conventional reasoning (DIT-N2 Scores) across groups. Using BOLD fMRI during the BART to examine evoked brain activity accompanying decision making under increasing risk, we also found greater activity in the VS in the post-con compared to the pre-con + con group. Our results provide converging evidence that individuals at a higher level of moral reasoning are characterized by increased activation in the mesolimbic frontostriatal system, in particular in value-related reward-processing regions (VS and vmPFC). According to previous studies, the VS is a key region in the mesolimbic frontostriatal reward processing system and plays an important role in value-based decision making, reinforcement learning, and affective processing (i.e., processing for reward and punishment)^24, 46–49^. It has been demonstrated that increased VS activation is linked to reward anticipation and evaluation^21, 46, 48^.

The vmPFC also plays an important role in reward processing, responding to various hedonic stimuli, reinforcement, and reward preferences^50–52^. Subjective valuation of reward-related stimuli is an important factor that activates both VS and vmPFC regions. Taken together, these findings extend our prior work showing a link between moral reasoning and gray matter structural volume in vmPFC/sgACC^5^ by demonstrating increased neural activity in these regions, both at rest and during a decision making task. Although these results are exploratory, they provide new insights into the potential neural basis and underlying psychological processing mechanism of individual differences in moral development.

As we mentioned before, value representations and reinforcement learning are shared components of moral judgment and decision making. Cushman’s dual-system approach which emphasizes valuation of actions versus valuation of outcomes provides a useful framework to understand diverse moral judgment and decision making^10^. From Cushman’s dual-system framework perspective (action-based value and outcome-based value) of moral judgment, activation differences in the VS and vmPFC/sgACC between the two DIT groups may reflect the potential importance of outcome valuation in moral reasoning development because the VS and vmPFC are more involved in outcome-based rather than action-based valuation in this framework. This is in line with Kohlberg’s theory that higher levels of reasoning do tend to focus on outcomes (i.e., the greatest good for the greatest number of people) rather than actions (i.e., this action is forbidden or permitted)^1^.

Additionally, based on the link between prosocial behaviors and high-level moral development^4, 14^, differences in prosocial motivation might be another important explanation for the link between brain activations and individual differences in moral development. Functional neuroimaging studies have consistently demonstrated the involvement of the VS and the vmPFC in both moral judgment^16, 22^ and prosocial behaviors such as charity and philanthropic activity^19, 26, 27^. Therefore, the current findings of the activation differences in the VS and vmPFC between two DIT groups may raise the potential importance of positive motivations towards others in moral reasoning development, rather than inhibition or selfish motives. Again, this is in line with Kohlberg’s theory that higher levels of moral reasoning tend to be promotion/other-focused (i.e., do it because it is right) rather than prevention/self-focused (i.e., do not do it because it is wrong).

There are other possible explanations for the links we observed, given that the vmPFC is involved in not only valuation but also affect regulation and social cognition (i.e., inferring others’ mental states and empathy for others)^53^. Thus, increased vmPFC/sgACC activity in the post-con group may be related to the ability to regulate emotional conflicts elicited by moral dilemmas or to the ability to infer others’ mental states (e.g., theory of mind and empathy). Notably, the AIS represents one’s own and others’ emotional states^54^ as well as being associated with aversion and harm-avoidance^55, 56^. Thus, the link between moral reasoning and IFG/AIS activity also points to a possible explanation in terms of sensitivity to aversion to harming others or empathic feelings to others.

In addition, a number of clinical studies have provided evidence that impairments in the brain’s reward system are associated with poor decision making, lack of empathy, and social inappropriateness. First, individuals with antisocial personality disorder and psychopaths, who are characterized by lower levels of moral reasoning than normal controls^12^, show structural and functional alterations in the VS^57^ and the vmPFC^58^. Psychopaths also exhibited reduced vmPFC activity during moral decision making^57^. Antisocial individuals also showed impaired VS activity in response to reward^57, 59^. Specifically, Gatzke-Kopp and colleagues^59^ found that normal controls had increased VS activity only during reward trials, while antisocial individuals with externalizing behavior disorders showed increased VS activity during both reward and non-rewarding trials, suggesting that antisocial individuals may be impaired in processing the omission of reward. Second, our previous findings regarding impulse control disorders (ICDs) in Parkinson’s Disease (PD) support the notion that the change in VS activation may underlie behavioral changes^29^. Dopaminergic therapy to treat PD can cause ICDs such as pathological gambling, excessive spending, and hypersexuality. Compared to PD patients without ICDs, PD patients with ICDs had significantly reduced activity in the VS during resting state and the BART, suggesting that abnormal behaviors observed in patients may result from diminished VS activity following dopamine treatment^29^. Taken together, all of the above-mentioned results have demonstrated that dysfunction of the VS and vmPFC are often linked to impulsivity and immoral behavior, which further supports our claim that the function differences in the VS and vmPFC regions are associated with the different levels of moral reasoning development.

We did not find any differences in risk taking on the BART between individuals at high-level post-conventional moral reasoning and individuals at the pre-conventional and conventional level of moral reasoning. This is consistent with the idea that people may view risky behaviors as a matter of personal choice instead of a moral issue^33, 34^, and that moral reasoning does not consistently predict risky behaviors^35–37^. However, at the neural response level, we do see that the post-conventional group was associated with higher VS activation during risky decision making. As this association was observed across all risk levels, it likely reflects intrinsic VS function rather than a VS response specific to risk.

We observed considerable individual differences in moral development levels in this relatively homogeneous and well-educated MBA group of subjects. It will be of great interest for future studies to know to what extent our DIT group differences depend on learned or in-born differences and to better understand why individuals with similar education may differ in their level of moral reasoning. Although the current results do not speak to this issue, we believe that both nurture (e.g., education, parental socialization, life experience etc.) and nature (e.g., biological or evolutionary basis, the innate capacities of the mind, the genetic basis, etc.) contribute to individual differences in moral development^60^. However, as a result of the correlative nature of this cross-sectional study, it is unclear whether brain function differences between the two groups are the cause or the result of differential levels of moral reasoning. Our study only documents differences in brain activation patterns associated with higher and lower levels of moral reasoning, but cannot explain the origin of those differences. Future research is needed to isolate the most formative socialization and educational experiences having an impact on moral development.

This study has some limitations which need to be addressed in future work. First, although other studies have demonstrated the predictive validity of the DIT for prosocial behaviors^4, 14^, we did not explicitly test whether post-conventional moral reasoning is associated with moral and prosocial behavior in our study. Future studies could also investigate the link between the neural correlates identified here and actual moral/prosocial behavior using moral reasoning or moral decision making tasks. Second, although our sample is relatively homogeneous in terms of age and level of education since these variables affect moral reasoning^2, 61^, unfortunately, we were not able to collect further education background information from our participants. For future research, it would be beneficial to know if individuals’ previous education background has any influence on their moral development. Third, previous studies have suggested that neural responsiveness in the VS and vmPFC may be modulated by mood states^62^. Although we did not assess participants’ mood states, this should be examined in further research. Fourth, we used the online version of the DIT-2^63^ to assess participants’ moral reasoning level. However, level of moral reasoning can also be defined according to other metrics, such as the prosocial moral reasoning objective measure (PROM), which is similar to the DIT-2, but centered on a caring or prosocial perspective rather than on a justice- or prohibition-oriented perspective^64^. Future studies could also characterize moral reasoning using this measure. In addition, although some evidence suggests that resting CBF measured by ASL fMRI primarily reflects trait-like brain function^65^, longitudinal studies are needed to determine the stability of CBF in the brain reward system. Finally, recent studies suggested that the VS may not be homogenous and subregions of the VS may play differential roles in reward learning and motivation^66–68^. Specifically, in the VS core, dopamine dynamics are consistent with learning-based theories (such as reward prediction error), whereas in the VS shell, dopamine dynamics are consistent with motivation-based theories (e.g., incentive salience)^66^. However, the limited spatial resolution of ASL and BOLD fMRI in this study does not allow us to localize the CBF and BOLD activation differences to more specific subregions of the VS, and it is unknown whether the observed differential VS activity between the two DIT groups reflects learning-based or motivation-based differences in moral reasoning development. Future imaging studies with much higher spatial resolution are needed to address this question.

In summary, we combined ASL perfusion and BOLD fMRI and examined both resting and task-induced brain activity associated with post-conventional moral reasoning in a relatively homogeneous cohort of 64 Wharton School MBA students. Individuals who reached the post-conventional moral reasoning level have increased resting CBF and task-induced activation in the VS and vmPFC/sgACC, which are key regions related to decision making and reward processing. This suggests that high-level moral reasoning is associated with increased activity in the frontostriatal system, both in task-dependent or task-independent states. These findings support the view that the development of morality is associated with measurable aspects of human brain function.

## Materials and Methods

### Participants

Sixty-four participants (35 males, average age = 27.11 ± 1.64) were involved in this study. These participants were selected from an overall sample of 730 students enrolled in a two-year Master of Business Administration (MBA) program based on their moral reasoning ability and are the same as those in our previous studies^5, 69^. All subjects completed an online version of the Defining Issues Test (DIT-2; refs 2, 63) and the Revised NEO Personality Inventory (NEO PI-R; ref. 70). The NEO PI-R, consisting of a 240 item questionnaire, was used to assess the Big Five personality traits: Openness to Experience, Conscientiousness, Extraversion, Agreeableness, and Neuroticism. For technical reasons, NEO PI-R data from two subjects were not available. Using the DIT-2, participants were stratified into two groups (see below): the post-conventional moral reasoning group (the post-con group) versus the pre-conventional and conventional group (the pre-con + con group). No subjects reported a history of head trauma or current psychiatric diagnosis, and subjects were free from any medications, including stimulant or serotonergic medications which can impact brain activation. Table 1 summarizes participants’ demographic information and behavioral performance on the BART. All study procedures adhered to the Declaration of Helsinki and were approved by the University of Pennsylvania Institutional Review Board (IRB). All subjects provided written informed IRB approved consent prior to participating in study procedures.

### Defining Issues Test, version 2 (DIT-2)

The online version of the DIT-2 measures the current level of moral reasoning for each participant^2, 63^. In this test, participants are required to read complex moral dilemmas (e.g., medically assisted suicide; http://ethicaldevelopment.ua.edu/dit-dilemmas/) and evaluate the relevance of different arguments to each dilemma. Based on cognitive schema preference, each participant can be assigned to a specific type level: Type 1—consolidated personal interests schema, Type 2—transitional personal interests schema, Type 3—transitional maintaining norms schema (personal interests schema is secondary), Type 4—consolidated maintaining norms schema, Type 5—transitional maintaining norms schema (post-conventional schema is secondary), Type 6—transitional post-conventional schema, and Type 7—consolidated post-conventional schema. In addition to the schema type, the DIT-2 provides a metric score (among other scores) reflecting the extent to which a person prefers post-conventional moral thinking (N2 score).

In this study, participants’ responses were scored by the Center for the Study of Ethical Development at the University of Alabama (http://ethicaldevelopment.ua.edu/), the publisher of the DIT-2. We divided participants into two levels based on the above-mentioned schema preferences: participants with post-conventional schema preference (Types 6 and 7) versus participants with personal interest and maintaining norms schemas preference (Types 1 to 5).

### Image data acquisition

All images were acquired on a Siemens 3.0 T Trio whole-body scanner (Siemens AG, Erlangen, Germany). ASL perfusion images were acquired using a pseudo continuous ASL (pCASL) sequence with a standard transmit/receive head coil for perfusion fMRI scans^71^. During the resting scan, participants were instructed to keep eyes open, and look at the fixation in the center of the screen. Acquisition parameters were as follows: repetition time (TR) = 4 s, echo time (TE) = 17 ms, labeling time = 1.5 s, delay time = 1.0 s, flip angle (FA) = 90°, field of view (FOV) = 220 × 220 mm, matrix = 64 × 64, slice thickness = 6 mm, inter-slice gap = 1.5 mm, and 4 minutes with 60 acquisitions. A standard echo-planar imaging (EPI) sequence was used to acquire BOLD fMRI data while participants performed the BART (TR = 1.5 s, TE = 24 ms, FOV = 220 × 220 mm, matrix = 64 × 64, 25 interleaved axial slices with 5 mm thickness, and 240 acquisitions). High-resolution anatomic images were also obtained using a standard 3D Magnetization Prepared Rapid Acquisition Gradient Echo (MPRAGE) sequence (TR = 1620 msec, TE = 3 msec, FA = 15°, 160 contiguous slices, voxel size = 1 × 1 × 1 mm).

### ASL resting CBF analysis

Data analysis was performed using SPM8 (www.fil.ion.ucl.ac.uk/spm/) and ASL Data Processing Toolbox, ASLtbx (https://cfn.upenn.edu/perfusion/software.htm)^72^. For each subject, functional images were realigned to correct for head motion and coregistered with the anatomical image. Perfusion weighted image series were then generated by pairwise subtraction of the label and control images, followed by conversion to absolute CBF image series based on a single compartment continuous arterial spin labeling (CASL) perfusion model^73^. For each subject, one mean resting CBF image was generated, normalized to the Montreal Neurological Institute (MNI) template, smoothed, and then entered into the whole brain voxel-wise analysis using a general linear model (GLM). A two-sample t-test was conducted to examine the CBF difference between the two groups at whole-brain level. Openness and Neuroticism were included in the GLM as nuisance covariates. Statistical significance threshold was set at cluster-level FWE-corrected *p* < 0.01 (cluster size >100) and at voxel-level uncorrected *p* < 0.005. For subsequent analysis, functional ROIs (including VS, vmPFC/sgACC, and IFG/AIS) were defined by setting a voxel-level uncorrected *p* < 0.001 and cluster size >50 to separate as single clusters. Then, partial correlation analysis (controlling for Openess and Neuroticism) was performed to test the association between individuals’ DIT-N2 scores and regional CBF values in three functional ROIs (VS, vmPFC/sgACC, and IFG/AIS) mentioned above based on our hypotheses. The distributions of these variables were confirmed to be normally distributed based on the Kolmogorov-Smirnov and Shapiro-Wik tests (all *p* > 0.1) using SPSS 22.

### BART and BOLD fMRI analysis

The BART was used to measure brain activation during risky decision making. The BART task and detailed fMRI analysis are described in Supplementary Materials. The balloon stimuli were categorized based on parametric risk level associated with the number of balloon inflations. The maximum balloon inflations per trial was 9 across all participants; therefore, 9 contrasts were established in the GLM model – BOLD values associated with each risk level (i.e., each inflation pump, 1–9) were calculated from the first to the ninth inflation. Using MarsBaR toolbox (http://marsbar.sourceforge.net/), parametric estimate values at each risk level were extracted from the same VS and vmPFC/sgACC functional ROIs defined from resting CBF analysis, which were described above. To further confirm our current finding, additional analyses were conducted using an a priori ROI identified by a previous meta-analysis of subjective value^24^.

## Electronic supplementary material

Supplementary Information

## References

[1] Kohlberg, L. Essays on moral development: Vol. 2. The psychology of moral development: Moral stages, their nature and validity (1984).

[2] Rest JR, Narvaez D, Thoma SJ, Bebeau MJ (1999). DIT2: Devising and Testing a Revised Instrument of Moral Judgment. J. Educ. Psychol..

[3] Walker LJ (2004). Progress and prospects in the psychology of moral development. Merrill. Palmer. Q..

[4] Rest, J. R. Moral development: Advances in research and theory (1986).

[5] Prehn K (2015). Neural Correlates of Post-Conventional Moral Reasoning: A Voxel-Based Morphometry Study. PLoS One.

[6] Cáceda R, James GA, Gutman DA, Kilts CD (2015). Organization of intrinsic functional brain connectivity predicts decisions to reciprocate social behavior. Behav. Brain Res..

[7] Crockett MJ (2013). Models of morality. Trends Cogn. Sci..

[8] Tobler PN, Kalis A, Kalenscher T (2008). The role of moral utility in decision making: an interdisciplinary framework. Cogn. Affect. Behav. Neurosci..

[9] Miller RM, Hannikainen IA, Cushman FA (2014). Bad Actions or Bad Outcomes? Differentiating Affective Contributions to the Moral Condemnation of Harm. Emotion.

[10] Cushman F (2013). Action, outcome, and value: a dual-system framework for morality. Personal. Soc. Psychol. Rev..

[11] Blair RJR (1997). Moral reasoning and the child with psychopathic tendencies. Pers. Individ. Dif..

[12] Blair RJ (1995). A cognitive developmental approach to mortality: investigating the psychopath. Cognition.

[13] Camerer, C. Behavioral game theory: Experiments in strategic interaction. (Princeton University Press, 2003).

[14] Thoma SJ, Rest JR, Barnett R (1986). Moral judgment, behavior, decision making, and attitudes. Moral Dev. Adv. Res. theory.

[15] Gummerum M, Keller M, Takezawa M, Mata J (2008). To give or not to give: Children’s and adolescents’ sharing and moral negotiations in economic decision situations. Child Dev..

[16] Heinzelmann N, Ugazio G, Tobler PN (2012). Practical implications of empirically studying moral decision-making. Front. Neurosci..

[17] Prehn, K. & Heekeren, H. R. In *Empirically Informed Ethics: Morality between Facts and Norms* 137–157 (Springer, 2014).

[18] Zahn, R., de Oliveira-Souza, R., Krueger, F. & Grafman, J. M. J. Opinion: the neural basis of human moral cognition. (2005). 10.1038/nrn1768 16276356

[19] Hare TA, Camerer CF, Knoepfle DT, Rangel A (2010). Value Computations in Ventral Medial Prefrontal Cortex during Charitable Decision Making Incorporate Input from Regions Involved in Social Cognition. J. Neurosci..

[20] Izuma K, Saito DN, Sadato N (2008). Processing of Social and Monetary Rewards in the Human Striatum. Neuron.

[21] Kable JW, Glimcher PW (2007). The neural correlates of subjective value during intertemporal choice. Nat. Neurosci..

[22] Shenhav A, Greene JD (2010). Moral judgments recruit domain-general valuation mechanisms to integrate representations of probability and magnitude. Neuron.

[23] Van der Meer MAA, Redish AD (2011). Ventral striatum: A critical look at models of learning and evaluation. Curr. Opin. Neurobiol..

[24] Bartra O, McGuire JT, Kable JW (2013). The valuation system: A coordinate-based meta-analysis of BOLD fMRI experiments examining neural correlates of subjective value. Neuroimage.

[25] O’Doherty JP, Cockburn J, Pauli WM (2017). Learning, Reward, and Decision Making. Annu. Rev. Psychol..

[26] Fehr E, Rockenbach B (2004). Human altruism: Economic, neural, and evolutionary perspectives. Curr. Opin. Neurobiol..

[27] Harbaugh WT, Mayr U, Burghart DR (2007). Neural Responses to Taxation and Voluntary Giving Reveal Motives for Charitable Donations. Science (80.)..

[28] Rao H, Korczykowski M, Pluta J, Hoang A, Detre JA (2008). Neural correlates of voluntary and involuntary risk taking in the human brain: an fMRI Study of the Balloon Analog Risk Task (BART). Neuroimage.

[29] Rao H (2010). Decreased ventral striatal activity with impulse control disorders in Parkinson’s disease. Mov. Disord..

[30] Schonberg, T. *et al*. Decreasing ventromedial prefrontal cortex activity during sequential risk-taking: An FMRI investigation of the balloon analog risk task. *Front. Neurosci*. 1–11 (2012). 10.3389/fnins.2012.00080 PMC3366349 22675289

[31] Weber MJ, Messing SB, Rao H, Detre JA, Thompson-Schill SL (2014). Prefrontal transcranial direct current stimulation alters activation and connectivity in cortical and subcortical reward systems: A tDCS-fMRI study. Hum. Brain Mapp..

[32] Kuther TL, Higgins-D’Alessandro A (2000). Bridging the gap between moral reasoning and adolescent engagement in risky behavior. J. Adolesc..

[33] Nucci L, Guerra N, Lee J (1991). Adolescent judgments of the personal, prudential, and normative aspects of drug usage. Dev. Psychol..

[34] Killen M, Leviton M, Cahill J (1991). Adolescent reasoning about drug use. J. Adolesc. Res..

[35] Berkowitz, M., Guerra, N. & Nucci, L. Sociomoral development and drug and alcohol abuse. *Handb. moral Behav. Dev. Vol. 3 Appl*. (1991).

[36] Berkowitz MW (1995). Assessing how adolescents think about the morality of substance use. Drugs Soc..

[37] Bush DF, Power C, Alterman AI, Connolly R (1981). Moral reasoning in alcoholics and addicts: structure vs content. Percept. Mot. Skills.

[38] Haan N, Stroud J, Holstein C (1973). Moral and ego stages in relationship to ego processes: A study of ‘hippies’. J. Pers..

[39] Mohr PH, Sprinthall NA, Gerler ER (1987). Moral reasoning in early adolescence: Implications for drug abuse prevention. Sch. Couns..

[40] Lejuez CW (2002). Evaluation of a behavioral measure of risk taking: The Balloon Analogue Risk Task (BART). J. Exp. Psychol. Appl..

[41] Hunt MK, Hopko DR, Bare R, Lejuez CW, Robinson EV (2005). Construct validity of the balloon analog risk task (BART) associations with psychopathy and impulsivity. Assessment.

[42] Lejuez CW (2003). The balloon analogue risk task (BART) differentiates smokers and nonsmokers. Exp. Clin. Psychopharmacol..

[43] Lejuez CW, Aklin WM, Zvolensky MJ, Pedulla CM (2003). Evaluation of the Balloon Analogue Risk Task (BART) as a predictor of adolescent real-world risk-taking behaviours. J. Adolesc..

[44] Li N (2013). Resting-state functional connectivity predicts impulsivity in economic decision-making. J. Neurosci..

[45] Di Martino A (2008). Functional connectivity of human striatum: a resting state FMRI study. Cereb. cortex.

[46] Delgado MR, Nystrom LE, Fissell C, Noll DC, Fiez JA (2000). Tracking the Hemodynamic Responses to Reward and Punishment in the Striatum Tracking the Hemodynamic Responses to Reward and Punishment in the Striatum. J. Neurophysiol..

[47] Knutson B, Gibbs SEB (2007). Linking nucleus accumbens dopamine and blood oxygenation. Psychopharmacology (Berl)..

[48] Knutson B, Katovich K, Suri G (2014). Inferring affect from fMRI data. Trends Cogn. Sci..

[49] Zald DH (2004). Dopamine transmission in the human striatum during monetary reward tasks. J. Neurosci..

[50] Kringelbach M (2005). L. the Human Orbitofrontal Cortex: Linking Reward To Hedonic Experience. Nat. Rev. Neurosci..

[51] Rushworth MFS, Noonan MP, Boorman ED, Walton ME, Behrens TE (2011). Frontal Cortex and Reward-Guided Learning and Decision-Making. Neuron.

[52] Rogers RD (2004). Distinct portions of anterior cingulate cortex and medial prefrontal cortex are activated by reward processing in separable phases of decision-making cognition. Biol. Psychiatry.

[53] Delgado MR (2016). Viewpoints: Dialogues on the functional role of the ventromedial prefrontal cortex. Nat. Publ. Gr..

[54] Rilling JK, Sanfey AG (2011). The neuroscience of social decision-making. Annu. Rev. Psychol..

[55] Paulus MP, Rogalsky C, Simmons A, Feinstein JS, Stein MB (2003). Increased activation in the right insula during risk-taking decision making is related to harm avoidance and neuroticism. Neuroimage.

[56] Christopoulos GI, Tobler PN, Bossaerts P, Dolan RJ, Schultz W (2009). Neural correlates of value, risk, and risk aversion contributing to decision making under risk. J. Neurosci..

[57] Glenn AL, Raine A, Schug RA (2009). The neural correlates of moral decision-making in psychopathy. Mol. Psychiatry.

[58] Yang Y, Raine A (2009). Prefrontal structural and functional brain imaging findings in antisocial, violent, and psychopathic individuals: a meta-analysis. Psychiatry Res. Neuroimaging.

[59] Gatzke-Kopp LM (2009). Neurological correlates of reward responding in adolescents with and without externalizing behavior disorders. J. Abnorm. Psychol..

[60] Killen M, Smetana JG (2015). Origins and Development of Morality. Handb. child Psychol. Dev. Sci..

[61] Bebeau, M. J. & Thoma, S. J. Guide for DIT-2. *Minneap. Cent. Study Ethical Dev. Univ. Minnesota* (2003).

[62] Price JL, Drevets WC (2010). Neurocircuitry of mood disorders. Neuropsychopharmacology.

[63] Xu Y, Iran-Nejad A, Thoma SJ (2007). Administering defining issues test online: Do response modes matter. J. Interact. Online Learn..

[64] Eisenberg-Berg N (1979). Development of children’s prosocial moral judgment. Dev. Psychol..

[65] Hermes M (2009). Latent state–trait structure of cerebral blood flow in a resting state. Biol. Psychol..

[66] Saddoris MP, Cacciapaglia F, Wightman RM, Carelli RM (2015). Differential Dopamine Release Dynamics in the Nucleus Accumbens Core and Shell Reveal Complementary Signals for Error Prediction and Incentive Motivation. J. Neurosci..

[67] Salgado S, Kaplitt MG (2015). The nucleus accumbens: A comprehensive review. Stereotact. Funct. Neurosurg..

[68] Ito R, Hayen A (2011). Opposing Roles of Nucleus Accumbens Core and Shell Dopamine in the Modulation of Limbic Information Processing. J. Neurosci..

[69] Jung WH (2016). Moral competence and brain connectivity: A resting-state fMRI study. Neuroimage.

[70] Costa, P. T. & McCrae, R. R. Neo PI-R professional manual. (1992).

[71] Zhu S, Fang Z, Hu S, Wang Z, Rao H (2013). Resting State Brain Function Analysis Using Concurrent BOLD in ASL Perfusion fMRI. PLoS One.

[72] Wang Z (2008). Empirical optimization of ASL data analysis using an ASL data processing toolbox: ASLtbx. Magn. Reson. Imaging.

[73] Wang J (2005). Amplitude-modulated continuous arterial spin-labeling 3.0-T perfusion MR imaging with a single coil: feasibility study. Radiology.

