# Supplementary material for: Post-conventional moral reasoning is associated with increased ventral striatal activity at rest and during task
Source: Sci Rep. 2017 Aug 2;7:7105. doi: 10.1038/s41598-017-07115-w (PMC5541100; doi:10.1038/s41598-017-07115-w)
Supplement: Supplementary file 1 — Supplementary Information [file 41598_2017_7115_MOESM1_ESM.pdf]

Supplementary Information

Post-conventional moral reasoning is associated with increased ventral striatal activity at rest and during task

Zhuo Fang[a,b,1], Wi Hoon Jung[c,1], Marc Korczykowski[b,d], Lijuan Luo[a,d], Kristin Prehn[e], Sihua Xu[a,b], John A. Detre[b], Joseph W. Kable[c], Diana C. Robertson[d,]*, and Hengyi Rao[a,b,]*

[a]Laboratory of Applied Brain and Cognitive Sciences, Shanghai International Studies University, Shanghai, China; [b]Center for Functional Neuroimaging, Department of Neurology, University of Pennsylvania, Philadelphia, United States of America; [c]Department of Psychology, University of Pennsylvania, Philadelphia, United States of America ; [d]Department of Legal Studies & Business Ethics, The Wharton School, University of Pennsylvania, Philadelphia, United States of America ; [e]Department of Neurology & NeuroCure Clinical Research Center, Charité Universitätsmedizin Berlin, Berlin, Germany

[1]Z.F. and W.H.J contributed equally to this work.

*Corresponding authors:

Hengyi Rao, PhD

Center for Functional Neuroimaging, Department of Neurology, University of Pennsylvania, 3W Gates, 3400 Spruce Street, Philadelphia, PA 19104, USA

Phone: (01) 215-746-2470, Email: hengyi@mail.med.upenn.edu.

or

Diana C. Robertson, PhD

Department of Legal Studies & Business Ethics, The Wharton School, University of Pennsylvania, 671 Huntsman Hall, 3730 Walnut Street, Philadelphia, PA, 19104, USA

Phone: (01) 215-898-5919, Email: robertsd@wharton.upenn.edu

Methods

BART procedure

The BART is a sequential risk-taking task (refer to 22 for detail explanation). During the task, participants were requested to sequentially press a button to inflate a balloon that could either grow larger or explode. Larger balloons were associated with greater risk of explosion and larger monetary rewards. Participants were repeatedly given two options: (i) to press the right button to continue inflating the balloon or (ii) to press the left button to discontinue inflation and collect the wager for the current balloon. If participants chose to stop inflation, they won the wager and the amount of reward was added to the cumulative earnings. If participants chose to continue inflation and the balloon exploded, they lost the wager and the lost monetary amount was subtracted from the cumulative earnings as the penalty. In order to encourage participants to make multiple inflation attempts for one balloon, the wager size and probability of explosion both monotonically increased with the number of inflations. The maximum number of inflations participants could make for each balloon was 12. From the smallest balloon to the largest balloon, the probability of explosion monotonically increased from 0 to 89.6%, and the wager increased from $0 to $32.08. The speed of inflation during the task was controlled by a small circle cue. That is, only when the color of the cue was green, participants could press a button to continue or discontinue inflation. The cue immediately turned red after inflation, and then turned green again after 1.5-2.5 seconds to indicate the next inflation. The value of wages corresponding to the various balloon sizes and the cumulative earnings for the tasks were explicitly displayed underneath the balloon stimuli, whereas the maximum number of inflations and the exact probability of explosion associated with a given inflation were unknown to participants.

BOLD fMRI analysis at whole brain level

We used SPM8 (www.fil.ion.ucl.ac.uk/spm/) for imaging data preprocessing and analyses. For each subject, functional images were realigned to correct head motion, corrected for slice acquisition time differences, and coregistered with the anatomical image. Data were then normalized to the standard MNI brain template with a 2×2×2 mm^3 voxel size, smoothed with 8 mm FWHM Gaussian kernel, and entered into a voxel-wise analysis using general linear model (GLM). A high-pass filter with a cut-off at 128 seconds was used to remove low frequency fluctuations.

Preprocessed data were modeled in an event-related design using a standard hemodynamic response function (HRF) with time derivative. For whole brain analysis, the model included

three regressors representing three types of events after pressing a button: (i) balloon inflation (i.e., onset of a larger balloon), (ii) a win outcome, and (iii) a loss outcome. We calculated brain activations with parametric risk level. The probability (i.e., the risk) of explosion for each balloon, orthogonalized by mean central correction, was entered into the model as a linear parametric modulation of the balloon inflation regressor. For each subject, a contrast of risk was defined to examine brain activations covaried with the parametric level of risk. One-sample t-tests were used to generate within-group activation maps. Two-sample t-tests were used to determine group differences between the two groups. Statistical significance thresholds were set at cluster-level FWE-corrected $p<0.05$ and at voxel-level $p<0.005$ uncorrected.

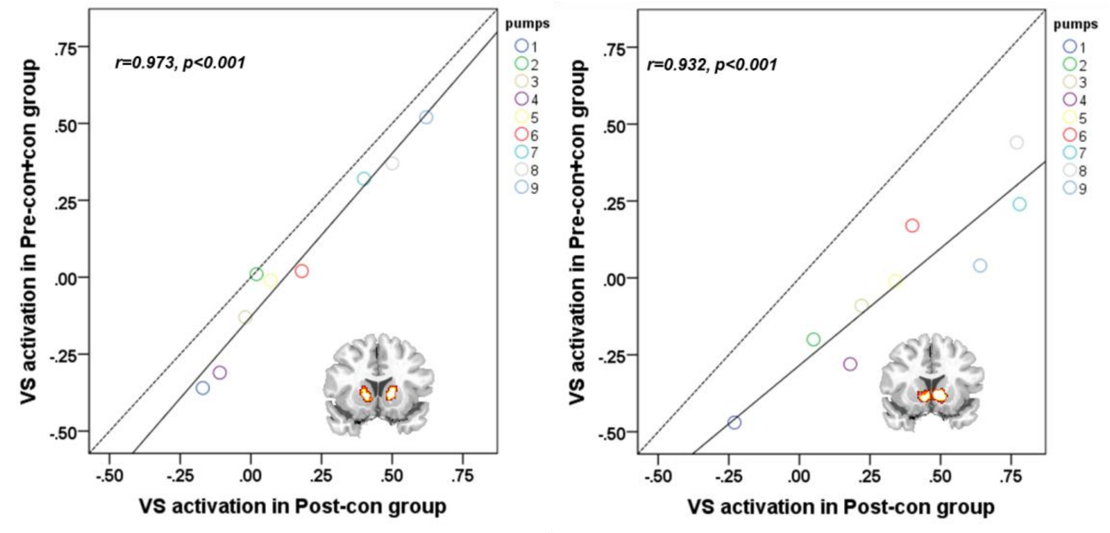

Figure S1. Correlation between BOLD response amplitudes in the VS of the post-con group and those of the pre-con+con group during the BART. (A) data were extracted from the functional VS ROI identified from CBF comparisons. (B) data were extracted from the independent VS ROI defined from a previous meta-analysis study (24). Each circle represents the average BOLD response amplitudes across trials and subjects for each risk level. The risk level evoked nearly identical responses change patterns between the two groups, suggesting that the increased VS activation in the post-con group is inherent but not specific to a certain risk level.

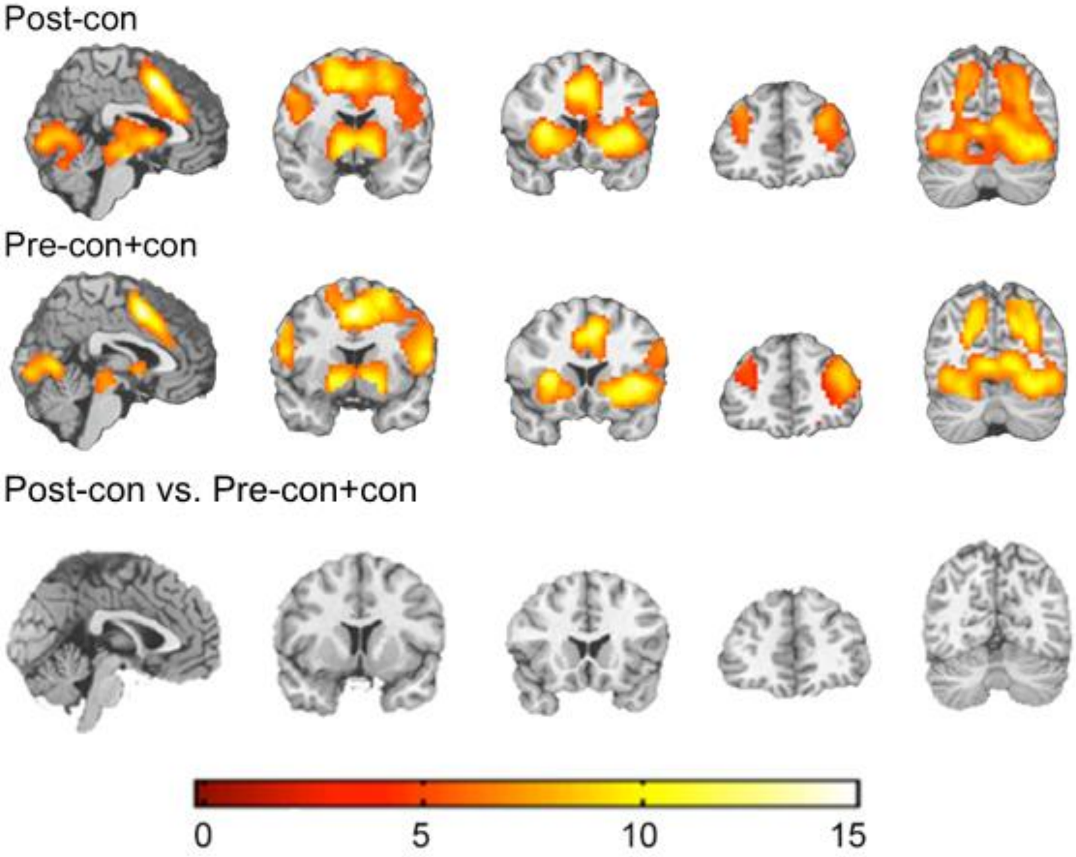

Figure S2. Whole brain activation co-varied with the parametric risk level during the BART. In both post-con (top) and pre-con+con groups (middle), increasing risk level was associated with robust activations in mesolimbic-frontal regions, including the midbrain, ventral and dorsal striatum, anterior insula, dorsal lateral prefrontal cortex, and anterior cingulate/media frontal cortex, as well as the activation in visual pathway regions. No activation differences were found between the two DIT groups (bottom).

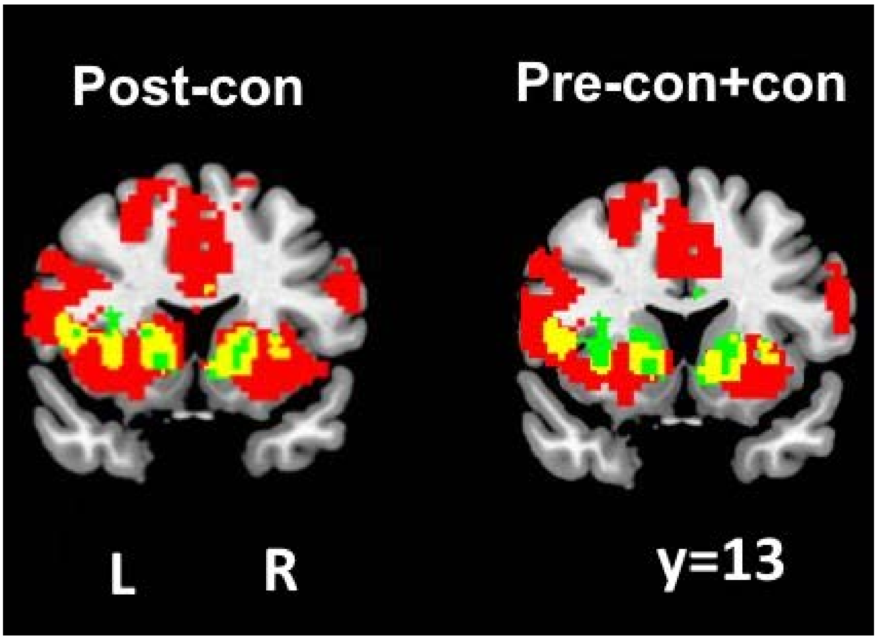

Figure S3. Overlap of VS activation across CBF and BOLD activation in two DIT groups during the BART. VS region showing CBF differences (Post-con vs. Pre-con+con) (Yellow) overlapped substantially with the VS region showing activation in the pre-conventional + conventional group and the post-conventional group during the BART (Red). Green regions are the overlapping of Yellow and Red.
